# Helping women transition out of sex work: study protocol of a mixed-methods process and outcome evaluation of a sex work exiting program

**DOI:** 10.1186/s12905-020-01086-3

**Published:** 2020-10-09

**Authors:** Martine Shareck, Pearl Buhariwala, Maha Hassan, Patricia O’Campo

**Affiliations:** 1 Département des sciences de la santé communautaire, Université de Sherbrooke, 3001 12è Avenue, Sherbrooke, Québec J1H 5H3 Canada; 2 Centre de recherche du Centre Hospitalier de l’Université de Sherbooke, Sherbrooke, QC Canada; 3 Institut Universitaire de Première Ligne en Santé et Services Sociaux, CIUSSS de l’Estrie-CHUS, Sherbrooke, QC Canada; 4 MAP Centre for Urban Health Solutions, St. Michael’s Hospital, Toronto, ON Canada; 5 Dalla Lana School of Public Health, University of Toronto, Toronto, ON Canada

**Keywords:** Sex work, Evaluation, Process, Implementation, Outcome, Critical time intervention, Social determinants of health

## Abstract

**Background:**

For women who want to, exiting sex work can be challenging. Numerous programs strive to help women wanting to exit sex work and secure alternative sources of income by providing targeted support at key moments during the transition, yet few of those initiatives are rigorously evaluated. In 2017 “Exit Doors Here”, a 9-month sex work exiting program based on the critical time intervention (CTI) approach, was developed to provide wrap-around support services (e.g., health, addiction, housing, education, and employment supports) to women wishing to transition towards exiting sex work.

**Methods:**

We present the design of an evaluation study of Exit Doors Here which combines quantitative and qualitative methods to assess participant recruitment and retention into the program, program fidelity, and relationships with service providers (process evaluation), as well as progress made by participants in terms of strengthening their social support networks and moving closer to achieving their housing, pre-employment (i.e., educational, training and volunteering), and income-related goals, as well as their involvement in sex work (outcome evaluation). Each year for 4 years, between 25 and 30 Exit Doors Here clients will be invited to complete an interviewer-administered questionnaire at the beginning and after completing the program, and to share data from their CTI charts and related documentation. Once a year, program staff and peer workers will be interviewed, and service providers will be surveyed.

**Discussion:**

Conducting a formative (process) evaluation will allow us to inform program implementation and improve program delivery early on for maximum benefit. The summative (outcome) evaluation will provide much needed evidence on the effectiveness of CTI in supporting a traditionally underserved population to achieve the housing, pre-employment and income-related goals they value, and their progress towards reducing their involvement in, and eventually exiting, sex work.

## Background

Between 2009 and 2014 in Canada, police services reported approximately 16,000 prostitution-related offences, making it a leading cause for law enforcement at the street level [1]. Of these, 4000 were reported in Ontario, the highest across Canada [2]. In Ontario, the majority of reported human trafficking cases, which is one of the fastest-growing crimes worldwide, involve sexual exploitation [3]. The Toronto east downtown core area is particularly well known for street prostitution where hundreds of sex workers provide prostitution related services on the streets and in the city’s strip clubs, massage parlours and underground brothels [4]. For women who want to, exiting sex work can be complicated by multi-traumatic symptoms and challenges related to addictions, physical and mental health problems, legal matters, housing issues, and lack of employment skills [5–9]. Dalla (2006) identified economic instability, mental health challenges, and existing relationships with significant others as barriers to women’s successful exit out of sex work, while facilitating factors included community, familial and peer support as well as caring for children. To be effective at supporting women’s efforts to exit sex work, interventions should be developed to address these issues in a comprehensive and coordinated fashion [8–13], however, few such interventions exist [14].

Existing interventions and programs that support exiting sex work may take on various forms. Residential treatment centres offer intensive time-limited psychoeducational programs targeting issues such as mental health, substace abuse or trauma [9, 11, 14, 15], while diversion programs involve a collaboration between different community services (e.g., law enforcement, social services, and community members) to provide women with addiction treatment, mental health services, and housing or employment services, and support them in avoiding jail time and eventually exit sex work [16, 17]. In a similar fashion, case management involves outreach workers linking their target clientele with community services including housing, mental health and substance abuse treatment services [10].

To date, very few interventions supporting women who want to exit sex work have been rigorously evaluated. Little evidence thus exists around how such interventions could be successfully implemented and how effective they might be in reaching their objectives [11]. While some psychoeducational interventions, such as the community treatment ESUBA: Women Helping Women Turn Abuse Around program, have been found to be effective in managing trauma symptoms among sex workers, their aim was not to support women’s exiting process per se; they were therefore not evaluated for their impacts on women’s involvement in, or exit from, sex work [11]. Similarly, a secondary analysis of a US-wide evaluation of substance-use disorder treatment programs found that the provision of ancillary medical, mental health, and psychosocial (e.g., employment seeking) services to women involved in sex work correlated with their successful exit 1 year after discharge [12]. However, the programs were not tailored to women involved specifically in sex work and their objectives were not to support their exiting process. Diversion programs have been suggested to be a cost-effective and holistic approach to reducing sex work recidivism (i.e., re-arrest), but they are often offered to first-time offenders who are young and new to criminalized behaviour [17], so it is unclear whether benefits would extend to a more diverse population of sex workers. Further limitations of these evaluation studies include low retention and follow-up rates, small sample sizes, qualitative methods mainly, and weak evaluation designs [18].

In order to address these gaps in evidence around ‘what works’ for supporting women seeking to exit sex work, and ‘how’ it works, we set out to conduct a process and outcome evaluation of Exit Doors Here, a program which was specifically developed to provide wrap-around support services to women who want to exit sex work. Our aims are to:

1. determine how program implementation is operating, and what components of the program might need adjustment to best fit the needs and realities of clients before being scaled up and implemented more widely, and.
2. assess the extent to which participation in the program is successful in helping women progress towards achieving their goals and transitioning out of sex work.

### Exit doors here: the program

Exit Doors Here is funded by Public Safety Canada, the Federal department which provides policy leadership, coordination and program support to prevent crime, enforce the law and rehabilitate offenders. The program is implememented by a Toronto-based non-profit organization which works with women to create meaningful changes in their lives, avoid conflict with the law and build a sustainable livelihood. This organization has recently seen a dramatic increase in the number of sex workers accessing their services for concerns surrounding legal matters, housing issues, addictions, and medical and mental health problems, and were thus favourably positioned to implement the program.

Exit Doors Here is a 9-month capacity-building program during which women who have expressed the desire to exit sex work closely work with a case manager to address one to three areas that they view as essential to their making a successful transition towards exiting. These areas may include housing, mental health, health and wellness, employment/vocational training/education, community and life skills, or friends and family. Focus areas for women who care for children or other family members include health and wellness, parenting and life skills, rent and entitlement management (e.g., social assistance benefits, child support), employment/vocational training/education, children’s service and school issues, and risky behaviours [19]. Throughout the program case managers work one-on-one with women to strengthen their support networks by connecting them with culturally-appropriate community-based service providers, peers and family members who will assume the primary role of support after the program is over. They also consolidate women’s skills development in an effort to support their self-confidence and employability, reduce their involvement in sex work and promote their successful transition out of sex work.

**Fig. 1:**
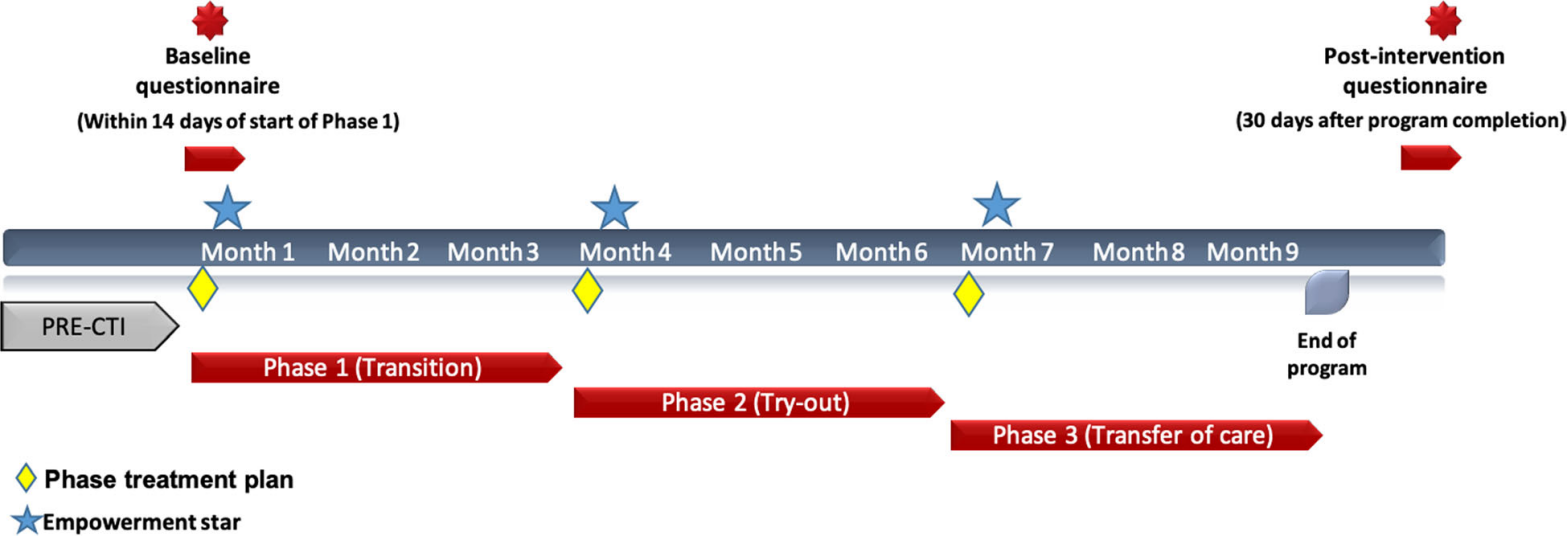
Timeline of CTI Phases and Data Collection for the Exit Doors Here Evaluation Study

The program is rooted in the Critical Time Intervention (CTI) model initially developed by Susser et al. (1997) to assist individuals in exiting homelessness [20]. The model has since been adapted for other populations such as victims of domestic violence [21, 22] and men leaving prison [23]. To our knowledge, only one research study on CTI among sex workers has previously been conducted [24]. The CTI model is based on the assumption that individuals’ successful and sustainable transition away from high risk lifestyles requires connections to long-term community-based supports. CTI is a time-limited intervention divided into three phases of 3 months each with decreased intensity over time (Fig. 1). Over three three-month phases, individuals work one-on-one with their case worker to co-develop a treatment plan based on their needs (Phase 1), try out the community supports set up for them (Phase 2), and see their care transferred from their CTI case manager to community services who they can work with in the long-term (Phase 3). A pre-CTI phase which has no time restriction can be built in to allow case managers to start building a rapport with their clients as they begin their transition. This model of intervention recognizes that exiting sex work is a staged process, that distinct cognitive and behavioral adjustments are needed to support behavioural change among populations facing multiple challenges, and that relapses and disengagement can occur and hinder exiting goals.

All female-identifying individuals who consider themselves either street-based, trafficked or non self-identified sex workers (i.e., women who do not identify as sex workers but nonetheless trade sex for money or other commodities) and want to exit sex work are eligible to enroll in Exit Doors Here. Additional program eligibility criteria include being 18 years or older, residing in the Greater Toronto Area, and experiencing at least two of the following: (i) having been, or being at risk of conflict with the law, (ii) lacking positive social support networks, (iii) substance use with negative impact, (iv) unemployment/underemployment/lack of employment skills, (v) lacking basic life skills, (vi) lacking personal safety at the hands of an abuser, and (vii) being homeless or at risk of homelessness.

In keeping with the CTI model, case managers only work with a small number of clients at a time. It is expected that between 25 and 30 women will enroll in Exit Doors Here each year, complete the intake process, and move on to other stages of the program, for a total of approximately 125 to 150 women assisted over the 5-year duration of the program (2017–2022). Recruitment into the program will be done through outreach on the streets and in drop-in centres and service agencies, internal referrals from the organization’s existing list of clients, and external referrals via partner organizations such as the Toronto Counter Human Trafficking Network. An outreach campaign targeting key public locations including public transit stations and information boards in health centres will also be performed. The program operates from two sites in the Greater Toronto Area: one downtown and one in the city’s east end.

### Exit doors here: process and outcome evaluation

#### Study aims and design

A mixed-methods process and outcome evaluation will be conducted by an independent team of researchers from the MAP Centre for Urban Health Solutions at St. Michael’s Hospital in Toronto. The following questions will be assessed:

Process evaluation questions:

- Q1. Are program recruitment strategies effectively reaching the target clients?
- Q2. Are clients being retained in the program?
- Q3. How closely did the program adhere to key components of CTI?
- Q4. Is the program meeting service providers’ needs and expectations?

Primary outcome evaluation questions:

- Q5. Did participants’ level of social support increase?
- Q6. Did participants advance their readiness to make progress on their housing goals?
- Q7. Did participants advance their readiness to make progress on their pre-employment goals (e.g., education, training, and volunteering**)**?
- Q8. Did participants advance their readiness to make progress on their income-related goals?
- Q9. Did participants’ involvement in sex work decrease?

Secondary outcome evaluation questions:

- Q10. Did participants progress on their chosen focus areas?
- Q11. Did participants’ level of readiness to make changes in their lives increase?
- Q12. Did participants’ awareness of community support programs and services increase?

Finding a control group for this group of program participants that would avoid the problem of selection is virtually impossible. Therefore, we use a pre-post design but will conduct a contribution analysis to strengthen the ability to make inferences about causality. A contribution analysis is a theory-based approach that can be used to infer causality based upon the contribution the program is making to the observed results, and to confirm or revise a theory of change. We will use the program’s theory of change and information about alternative explanations for our findings to confirm that the program was influential in bringing about the observed results, taking other influencing factors into consideration. Evidence to develop the contribution story will come from a combination of literature reviews and primary data collected among women, program staff, and partnering service providers [25].

## Methods

### Sample

The evaluation will access data from three groups of individuals: (i) women enrolled in the Exit Doors Here program; (ii) program staff and peers; (iii) partnering service providers.

All women enrolled in the Exit Doors Here program will be invited to take part in the evaluation study by completing a baseline and post-intervention questionnaire in-person or virtually (during the COVID-19 pandemic) with a trained research assistant and/or granting the evaluators access to their CTI charts and related documentation. We aim to recruit 25 to 30 women into the evaluation study each year, which is approximately 83–100% of all women enrolled in Exit Doors Here. We intend to recruit women within 2 weeks of their starting Phase 1 of Exit Doors Here. Enrolment in the program and the evaluation will occur on a rolling basis. All program staff and volunteers will be invited to take part in annual semi-directed interviews. Organizations providing services and support to program staff and clients will be invited to complete an annual online survey.

### Ethical considerations

The study protocol has been approved by the St. Michael’s Research Ethics Board (approval #18–215). Prior to data collection women will provide written or verbal informed consent, service providers will provide written consent, and program staff will provide verbal consent. Women will be compensated for their time.

### Data sources and tools

A number of data sources will be used as summarized in Table 1 and described in more details below.

**Table 1:** Data Sources to be Used in the Exit Doors Here Evaluation

Evaluation question	Main data source(s)
Q1. Are program recruitment strategies effectively reaching the target clients?	CTI client charts; baseline client questionnaire
Q2. Are clients being retained in the program?	Program manager’s annual reports to funder
Q3. How closely did the program adhere to key components of CTI?	CTI client charts; staff and volunteer interviews
Q4. Is the program meeting service providers’ needs and expectations?	Service provider survey
Q5. Did participants’ level of social support increase?	Baseline and post-intervention client questionnaires
Q6. Did participants advance their readiness to make progress on their housing goals?	Baseline and post-intervention client questionnaires; CTI client charts
Q7. Did participants advance their readiness to make progress on their pre-employment goals (e.g., education,training, and volunteering**)**?	Baseline and post-intervention client questionnaires; CTI client charts
Q8. Did participants advance their readiness to make progress on their income-related goals?	Baseline and post-intervention client questionnaires; CTI client charts
Q9. Did participants’ involvement in sex work decrease?	CTI client charts; post-intervention client questionnaire
Q10. Did participants progress on their chosen focus areas?	CTI client charts (empowerment stars)
Q11. Did participants’ level of readiness to make changes in their lives increase?	Baseline and post-intervention client questionnaires
Q12. Did participants’ awareness of support services increase?	Post-intervention client questionnaire

#### Baseline client questionnaire

The baseline questionnaire collects socio-demographic data, information about legal issues, satisfaction, goals, and challenges related to housing, employment, education and training, and financial situations (Additional file 1). To assess participants’ level of readiness to make changes in their lives, we will use the validated University of Rhode Island Change Assessment Scale. It includes 32 statements rated on a five-point Likert scale [26, 27] which can be combined into an overall “readiness to make changes” stage. Participants’ level of social support will be assessed with the validated 12-item Multidimensional Scale of Perceived Social Support [28]. The baseline questionnaire also includes six semi-structured interview questions pertaining to participants’ experience with, and reasons for, enrolling in Exit Doors Here, and what they hope to get out of the program.

#### Post-intervention client questionnaire

A post-intervention questionnaire will be administered approximately 1 month after women graduate from the program (Additional file 2). Similar questions as in the baseline questionnaire will be asked, in addition to nine questions assessing participants’ level of awareness and use of community services and supports after program completion [29]. Women will be asked to describe their current level of involvement in sex work as “not involved at all”, “involved rarely” or “involved frequently”. Open-ended questions will assess changes made by women to their housing, employment, education and training, and financial situations, and goals remaining to be achieved. Ten semi-structured interview questions will explore participants’ overall experience in the Exit Doors Here program.

#### Empowerment star

To assess participants’ progress on their selected CTI focus areas, we will rely on the Empowerment Star, an evidence-based tool originally developed for women who have experienced domestic violence but which can be adapted to other contexts [30]. The Empowerment Star is designed to support and measure change, and is underpinned by a person-centred, strengths-based and co-production approach to service delivery [30]. It is completed by clients together with their case manager, keeping clients’ perspectives and priorities front and center. At the beginning of each of the three CTI phases, clients rate the stage of change they feel they are at for all nine subscales of the Empowerment Star which overlap to some extent with CTI focus areas: safety, accommodation, support networks, legal issues, health and wellbeing, money, children, work and learning, and empowerment and self-esteem. This allows tracking of each individual’s journey of change through five stages: “Not ready for help”, “Accepting help”, “Believing”, “Learning and Rebuilding”, and “Independence and Choice”. The Empowerment Star is informed by the Transtheoretical Model (TTM) of Change which stipulates that intentional change in behaviour occurs as a process, not at a specific moment in time, and recognizes that individuals can transition back and forth between stages until they achieve stability [31, 32].

#### CTI client charts

Case managers complete several CTI charts documenting information about, and meetings with, their clients: (1) an *intake form* completed upon first meeting with the client to gather background and referral information; (2) an *assessment form* documenting clients’ strengths, risks, and needs; (3) a detailed *phase treatment plan* for each CTI phase based on clients’ goals; (4) *progress notes* documenting contacts between case managers, their clients and community and informal supports; (5) a *contact information form*; and (6) *closing notes* summarizing clients’ progress during the program. Data needed to answer evaluation questions will be systematically extracted from participants’ CTI charts.

#### CTI fidelity scale

The Dutch adaptation of the CTI fidelity scale will provide a quantitative assessment of two dimensions of program fidelity (i) compliance fidelity and (ii) competence fidelity [22]. Compliance fidelity measures the degree to which staff are practicing the key elements of CTI. It is measured by assessing the extent to which the program is being delivered to its intended structure and with skill and attention to the CTI model. Competence fidelity is assessed by measuring chart quality and completeness. The Dutch CTI fidelity scale comprises 12 items with one to five criteria each which can be rated as “fulfilled” (score of 1) or “not fulfilled” (score of 0) using data from participants’ CTI charts. Fidelity will be assessed in the first year of the program to provide staff with recommendations for change and improvement, and in each subsequent year for quality control purposes [33].

#### Staff interviews

Every year, Exit Doors Here staff and volunteers who provide direct services to clients will be invited to take part in a 20 to 30-min semi-structured interview conducted face-to-face or over the phone. The interviews will help identify factors which might influence program implementation, fidelity, and outcomes, and will inform the contribution analysis.

#### Service provider survey

A survey for service providers, jointly devised by the program and evaluation teams, will be used to assess whether service providers’ needs and expectations are fulfilled by the program and what could be done to improve on these fronts. The survey includes a mix of close-ended questions on 5-point Likert scales and open-ended questions.

### Data analysis

#### Process evaluation questions

To assess whether program recruitment strategies are effectively reaching target clients (Table 1, Q1), data from CTI charts will be used to compare the demographic characteristics and types of sex workers (street-based, trafficked, and non-identifying sex workers) recruited into the program to those of the target population. Client retention (Table 1, Q2) will be assessed every year and at the end of the program duration. In fact, although CTI has a strict “no drop-out” policy, it may happen that women do not complete the program. We will compare the number of women who enrolled in the program to those who graduated using information from the program director’s yearly reports to the funding agency.

To assess the extent to which the program is being implemented with fidelity to the CTI model (Table 1, Q3), data from participants’ CTI charts will be used to complete the CTI fidelity scale. In the first year, the Dutch version of the fidelity scale will be validated against data from a subset of women (*n* = 8) and results will be discussed with the program team. The fidelity scale may be adapted accordingly to better reflect the context of working with women attempting to exit sex work. Following this validation step, the adapted fidelity scale will again be completed every year using data from five randomly selected participants. Item-level fidelity ratings will be interpreted in light of a 5-point rating scale: program not implemented in line with CTI model, poorly implemented, fairly implemented, well implemented, and ideally implemented [22]. Each year, interviews with program staff and volunteers will complement the interpretation of fidelity scores to identify factors that may enable or constrain high fidelity in program delivery. Subsequent adjustments may be made to the program if needed [23]. Finally to assess if the program is meeting service providers’ needs and expectations (Table 1, Q4), responses to the service provider survey will be described in frequency tables or qualitatively summarized.

#### Primary outcome evaluation questions

To assess the change in participants’ level of social support (Table 1, Q5), we will compute the difference in overall mean scores across the 12 items from the Multidimensional Scale of Perceived Social Support [28] between post-intervention and baseline, and report the proportion of participants with an increase, decrease, or no change in perceived social support.

To assess if participants advanced their readiness to make progress on their housing, pre-employment, and income-related goals (Table 1, Q6, 7, and 8 respectively), data will be systematically extracted from participants’ closing notes in which case managers document the changes made, and progress achieved, by each participant, for each of these areas. Outcomes may include “submitted subsidized housing application”, “signed up for MyStartUp or H&R Bloc training program” or “secured Ontario Disability Support Program (ODSP)”. With these data we will categorize women according to whether they have progressed towards their initial goals. This is will be complemented by post-intervention questionnaire data in which women discuss facilitators and barriers to making progress (or not) on their initial goals.

Since exiting sex work is a process, it is unrealistic to expect that all clients will attain their long-term goal of exiting sex work within the limited timeframe of CTI. In fact, the stated goal of CTI is to “successfully link clients to supports who will eventually take over helping clients meet their long-term goals” [34]. To assess whether participants have reduced their involvement in sex work (Table 1, Q9), we will compute the proportion of women reporting being “involved rarely” and “not involved at all” in sex work in the post-intervention questionnaire.

#### Secondary outcome evaluation questions

Participants’ progress on their chosen CTI focus areas (Table 1, Q10) will be assessed by comparing their Empowerment Star ratings from the beginning of Phases 1 and 3 and reporting the proportion of women who, among those who will have chosen each of the nine Empowerment Star focus areas, will have “progressed at least one stage”, “remained at the same stage” or “regressed at least one stage” for each of the nine Empowerment Star areas.

To assess participants’ progress in terms of readiness to make changes in their lives (Table 1, Q11), we will follow the University of Rhode Island Assessment Scale guidelines to compute their overall “readiness to change” score and assign participants an overall stage of readiness to change: pre-contemplation, contemplation, action, and maintenance [27]. Stages will be compared between baseline and post-intervention assessments. We will report the proportion of participants who have moved forward on the continuum of stages, those who have remained at the same stage, and those who have moved backwards on the continuum of stages. Finally, to explore participants’ awareness and use of support services at the end of the program (Table 1, Q12), we will compile the proportion of participants reporting agreement with each of the nine statements tapping into this concept.

## Discussion

This process and outcome evaluation of the Exit Doors Here program will provide much needed evidence on the implementation of a critical time intervention to help women transition out of sex work. CTI has, to date, been conducted with well-circumscribed populations who, following discharge from institutional settings such as prisons, shelters or inpatient treatment facilities, were transitioning to community living [21, 22]. Few studies of CTI programs implemented with more transient populations recruited from the community and spread across a large geographic area have been performed. Results will provide insights on the program’s effectiveness in supporting a traditionally underserved population to achieve the housing, pre-employment (i.e., educational, training and volunteering) and income-related goals they value, and their progress towards reducing their involvement in, and eventually exiting, sex work. It will also shed light on clients’ and staff’s perspectives on the factors that may enable or constrain the successful implementation of such a program.

The evaluation study has several strengths. It was designed in close collaboration with the program team who contributed invaluable insights into the issues women may be facing and which should be documented, and helped with evaluation question formulation and interview question wording for increased understanding by participants. We will conduct both formative (process) and summative (outcome) evaluations, with the former done in the first 2 years to allow modifying and improving the program and its delivery for maximum benefit. We will also be assessing mid-term progress towards achieving goals by comparing empowerment stars from Phases 1 and 3, as well as longer-term progress from beginning to the end of program (with CTI closing notes) and 1 month later (with the post-intervention questionnaire). Combining qualitative and quantitative methods in both process and outcome evaluations will provide a comprehensive understanding of *if* the program is having the expected impacts and *how* it is achieving the observed effects. We are maximizing the number of women to be included in the evaluation study by aiming to recruit them as early as possible through both direct (attendance at weekly information sessions held for program enrolees) and indirect means (through case managers), which can also bolster participant engagement. Our reliance on both standardized data collection instruments is also a strength, along with our use of a combination of self-reported (questionnaires) and objective data (CTI charts) which allows for triangulation. Performing a contribution analysis is also a proven way of overcoming the limitation of not having a control group [25].

We anticipate a number of challenges and have devised solutions to address them as they arise. Women involved in sex work are known to be a hard-to-reach population. Despite relying on a combination of active and passive outreach and recruitment methods, the program, and consequently the evaluation study, are likely to only reach a subset of women, i.e., those who may already be empowered to make changes in their lives and succeed in their transition towards exiting sex work. These women may not be representative of the overall population of women involved in, and who wish to exit, sex work in Toronto. Many participants may also be transient and may not have a fixed place to live, which may increase the risk of attrition throughout the program and loss-to-follow up in the evaluation study since the post-intervention questionnaire is to be completed 1 month after program completion. To minimize this risk, we intend to stay in touch with women through various means: through direct contact, via their case workers, regular participation in information sessions, precise record keeping and updating of when and how to contact participants for follow-up, and sending a reminder before they finish the program [35]. Two peers involved in the program will also help support sustained participation and engagement in the program and evaluation [36]. Women can also go missing for a variety of reasons. We will interview women even if they can only be reached much later after they have completed the program since we value their participation and believe it is preferable to collect some information than none. The timing of baseline and follow-up data collection will be taken into account in the analyses. Another challenge is that women may not all consider what they do as “sex work”. After discussing with the program team, we have concluded that a straightforward question about women’s involvement in sex work may not be reliable. Therefore, we will probe for multiple expressions women use to refer to sex work such as “going out on dates”, “dancing in clubs for money”, “exchanging sexual acts for rent, food, clothing, or shelter”, “working in a massage parlour”, and “going out on dates at certain times, such as the end of the month” as a means to obtain more valid responses.

A limitation of the evaluation design is that exiting sex work, just like transitioning out of other challenging situations such as homelessness, is not a linear, straightforward process [8]: there may be multiple relapses before women exit sex work for good. Collecting only one follow-up relatively soon (i.e., 1 month) after program completion prevents us from assessing potential relapses. Multiple follow-ups over a longer time period would be useful to counter this situation in future studies [35]. However, the expectation of the program is that women will *progress* towards achieving their goals, and this is what the evaluation is ultimately assessing. Women indeed may need workplace and pre-employment skills in order to earn a living wage and transition out of sex work, and furthermore, they may need transportation and childcare assistance to maintain their employment. Evaluating program impacts on these intermediary steps will provide a better understanding of the sex work exiting process than focusing exclusively on the final endpoint of actual exit. Finally, Exit Doors Here is targeted at women who express the desire to exit sex work. As such, we acknowledge that evaluation results will not be generalizable to all female sex workers, some of whom may not be ready to exit sex work, which we respect.

## Conclusion

Evidence is needed on if and how CTI can help support women who wish to exit sex work successfully transition towards that goal. Findings from this evaluation study will help identify what the key active ingredients in Exit Doors Here are and how the program could be scaled up and applied to other contexts. At a community level, findings will support Toronto’s harm reduction objectives among sex workers, and offer insights into how collaboration among social justice, public health, education, housing, and legal agencies in the city can better collectively address women’s needs.

## Supplementary information

**Additional file 1.**

**Additional file 2.**

## Data Availability

The datasets that will be generated during the current study are stored at the MAP Center for Urban Health Solutions, St. Michael’s Hospital, Toronto. Anonymized databases are available from the corresponding author upon reasonable request and with permission from St. Michael’s Hospital.

## Abbreviation

CTI: Critical Time Intervention
