# Supplementary material for: Helping women transition out of sex work: study protocol of a mixed-methods process and outcome evaluation of a sex work exiting program
Source: BMC Womens Health. 2020 Oct 9;20:227. doi: 10.1186/s12905-020-01086-3 (PMC7545381; doi:10.1186/s12905-020-01086-3)
Supplement: Supplementary file 1 — Additional file 1. [file 12905_2020_1086_MOESM1_ESM.docx]

**CONFIDENTIAL**

**EXIT DOORS HERE PROGRAM EVALUATION**

**BASELINE QUESTIONNAIRE**

**Date:**

**Participant ID:**

**CONFIDENTIAL**

“Thank you again for agreeing to take part in this survey. We will be asking some questions to learn about your experience with the *Exit Doors Here* program. We will also ask you a few questions that will help us get an overall picture of the program’s participants. **Remember that there is no right or wrong answer, and you can skip any question you do not feel comfortable answering**. This survey should take about one hour to complete. If you want to take a break at any time, let me know and we will stop before starting again. Please be assured that your name will not be linked to any of the information you provide.

***Program Questions***

How long have you been a part of the Exit Doors Here program?

_________________________________

Do you know what phase of CTI [Critical Time Intervention] you are currently in?

________________________________

***Housing Situation & Goals***

“I am first going to ask you some questions about your housing situation and any plans you might have about your housing situation in the future”.

**H_Q01 What is your current living arrangement?**

☐Renting a house/apartment/condominium

☐Own a house/condominium

☐Renting a room in a house/apartment with roommates

☐Don’t have a place to live

☐Staying temporarily with family members or friends

☐Staying at an emergency shelter

☐Staying at a women’s hostel

☐Other (please specify)

☐Decline to answer

**H_Q02 How satisfied are you with your current living situation?**

☐Very satisfied

☐Satisfied

☐Somewhat satisfied

☐Neither satisfied nor dissatisfied (neutral)

☐Somewhat dissatisfied

☐Dissatisfied

☐Very dissatisfied

☐Decline to answer

**H_Q03 Do you have any plans related to your housing situation? (Check all that apply).**

☐I would like to move into a new apartment/home

☐I want my own place to live

☐I want to live in housing that I can afford

☐I do not want to live with my family members/roommates anymore

☐I want to feel safer in my home

☐I want to live in a safer neighbourhood

☐I want to move into an apartment/home that is more spacious

☐I need housing that is accessible for a physical disability

☐I want a place to live that I feel proud of

☐I want my landlord to fix the repairs needed in my apartment/home

☐I would like to live closer to my friends/family

☐I am satisfied with my current housing situation, I do not plan to change it

☐Other (please specify)

☐Decline to answer

**H_Q04 If you want to change your housing situation but can’t, what are the reasons?**

**(Check all that apply)**

☐I can’t afford to move/can’t find an affordable place to live

☐I am scared to leave my current situation

☐I need to keep living near a family member (e.g., a parent in a nursing home)

☐I need to live near my children’s school/daycare

☐I need to find a new job, so I can afford to move

☐I am awaiting subsidized housing/ benefits

☐I have experienced discrimination from potential landlords when trying to look for a new place to live

☐Other (please specify)

☐Decline to answer

***Employment, Educational & Financial Situation & Goals***

“I am now going to ask you questions about your employment and financial situation, as well as goals you might have for changes over the course of the Exit Doors Here program. You can skip any question you do not want to answer”.

**E_Q01 Are you employed?**

☐Yes

☐No **🡪 Skip to E_Q03**

☐Decline to answer

**E_Q02**  [**If employed**], how satisfied are you with your employment or work situation?

☐Very satisfied

☐Satisfied

☐Somewhat satisfied

☐Neither satisfied nor dissatisfied (neutral)

☐Somewhat dissatisfied

☐Dissatisfied

☐Very dissatisfied

☐Decline to answer

**E_Q03 What was your individual income last year, before tax?**

☐$15,000 or under

☐$15,001-$25,000

☐$25,001-$35,000

☐$35,001-$50,000

☐$50,001-$75,000

☐More than $75,000

☐Decline to answer

**E_Q04 Do you have any plans to change jobs or receive further education in the future?**

**If so, what are they? (Check all that apply)**

☐I want to find a job

☐I want to find a different job

☐I want to train or become certified in a different field of employment

☐I want to enroll in college/university

☐I am satisfied with my current arrangement, I do not plan to change it

☐Other (please specify)

☐Decline to answer

**E_Q05 If you want to change jobs or receive further education but can’t, what are the**

**reasons? (Check all that apply)**

☐I don’t know where to start looking for information

☐I don’t have proper means of transportation

☐I don’t have proper childcare

☐I don’t have the skills or education to make the change

☐I have health issues that keep me from it

☐I have a learning disability

☐I have a permanent physical disability

☐Other (please specify)

☐Decline to answer

**E_Q06 Do you have any financial plans for the future? (Check all that apply)**

☐I want to earn more money

☐I want to become financially independent

☐I want to apply for social assistance and/or disability support

☐I want to open a bank account

☐I want to pay off my debt

☐I am satisfied with my current financial arrangement, I do not plan to change it

☐Other (please specify)

☐Decline to answer

**E_Q07 If you want to change your financial situation but can’t, what are the reasons?**

**(Check all that apply)**

☐I’m having trouble finding a job

☐I don’t know where to start looking for information

☐I don’t have the required skills/ education

☐I have health issues

☐Other (please specify)

☐Decline to answer

***Legal Issues***

“I am now going to ask you some questions and there are no choices like in the previous questions. These questions are for us to learn about any legal issue(s) you might be having and how it impacts your life.”

**L_Q01 Do you have any legal issue(s) at the moment?**

(Prompt: Are you involved in any legal cases due to involvement of close family members? Examples of legal issues could be family court for child custody, or housing and tribunal, or working towards pardon, or diversion program, e.g. probation, volunteering, community health service, or witness to cases i.e. witness services)

**L_Q02 If so, what kind(s) of legal issue(s) do you have?**

**L_Q03 How do these legal issue(s) affect your life?**

***Social Support Networks (The Multidimensional Scale of Perceived Social Support (MSPSS))***

“I am now going to ask you some questions about the social support that you have in your life such as friends, family, neighbours etc. I am going to read 11 statements to you. Some of the statements you might agree with, and others you might not. I would like you to tell me on a scale of 1 to 7 if you: very strongly disagree; strongly disagree; mildly disagree; neutral; mildly agree; strongly agree; very strongly agree.” *(Interviewer hands Scale # 1 to the participant)*

| **Statement** | **Very Strongly Disagree** | **Strongly Disagree** | **Mildly Disagree** | **Neutral** | **Mildly Agree** | **Strongly Agree** | **Very Strongly Agree** |
| --- | --- | --- | --- | --- | --- | --- | --- |
| SS_01  There is a special person who is around when I am in need. | 1 | 2 | 3 | 4 | 5 | 6 | 7 |
| SS_02  There is a special person with whom I can share my joys and sorrows. | 1 | 2 | 3 | 4 | 5 | 6 | 7 |
| SS_03  My family really tries to help me. | 1 | 2 | 3 | 4 | 5 | 6 | 7 |
| SS_04  I have a special person who is a real source of comfort to me. | 1 | 2 | 3 | 4 | 5 | 6 | 7 |
| SS_05  My friends really try to help me. | 1 | 2 | 3 | 4 | 5 | 6 | 7 |
| SS_06  I can count on my friends when things go wrong. | 1 | 2 | 3 | 4 | 5 | 6 | 7 |
| SS_07  I can talk about my problems with my family. | 1 | 2 | 3 | 4 | 5 | 6 | 7 |
| SS_08  I have friends whom I can share my joys and sorrows with. | 1 | 2 | 3 | 4 | 5 | 6 | 7 |
| SS_09  There is a special person in my life that cares about my feelings. | 1 | 2 | 3 | 4 | 5 | 6 | 7 |
| SS_10  My family is willing to help me make decisions. | 1 | 2 | 3 | 4 | 5 | 6 | 7 |
| SS_11  I can talk about my problems with my friends. | 1 | 2 | 3 | 4 | 5 | 6 | 7 |

***Readiness for Self-Change (University Rhode Island Change Assessment Scal)***

“We are interested in knowing how ready you feel to make changes today. I am going to read you short statements. I would like you to tell me on a scale of 1 to 5 if you strongly disagree; disagree; are undecided; agree; strongly agree” with each statement. *(Interviewer hands Scale #2 to the participant)*

| **Statement** | **Strongly**  **Disagree** | **Disagree** | **Undecided** | **Agree** | **Strongly**  **Agree** |
| --- | --- | --- | --- | --- | --- |
| RC_01 As far as I am concerned, I don’t have any problems or situations in my life that I want to change. | 1 | 2 | 3 | 4 | 5 |
| RC_02 I think I might be ready for some self-change. | 1 | 2 | 3 | 4 | 5 |
| RC_03 I am doing something about the problem or situation that has been bothering me. | 1 | 2 | 3 | 4 | 5 |
| RC_04 It might be worthwhile to work on my problems or to change a situation in my life. | 1 | 2 | 3 | 4 | 5 |
| RC_05 As far as I am concerned, I don’t have any problems or negative situations that need changing. | 1 | 2 | 3 | 4 | 5 |
| RC_06 I am not the one with a problem, so it doesn’t make sense for me to consider changing. | 1 | 2 | 3 | 4 | 5 |
| RC_07 I am doing some work on my problems or taking steps to change a negative situation in my life. | 1 | 2 | 3 | 4 | 5 |
| RC_08 I have been thinking that I might want to change something about myself or a situation that I am in. | 1 | 2 | 3 | 4 | 5 |
| RC_09 I have been successful in working on my problem or changing a negative situation, but I’m not sure I can keep up the effort on my own. | 1 | 2 | 3 | 4 | 5 |
| RC_10 At times my situation or problem is difficult to deal with, but I am working on it. | 1 | 2 | 3 | 4 | 5 |
| RC_11 Trying to change is pretty much a waste of time for me. | 1 | 2 | 3 | 4 | 5 |
| RC_12 I’m hoping that I will be able to understand myself better. | 1 | 2 | 3 | 4 | 5 |
| RC_13 There is really nothing I need to change. | 1 | 2 | 3 | 4 | 5 |
| RC_14 I am really working hard to change. | 1 | 2 | 3 | 4 | 5 |
| RC_15 I have a problem, and I really think I should work on it. | 1 | 2 | 3 | 4 | 5 |
| RC_16 I’m not following through with what I have already changed as well as I had hoped, but I am not going to give up. | 1 | 2 | 3 | 4 | 5 |
| RC_17 Even though I’m not always successful in changing my situation or my problems, I am at least working on changing. | 1 | 2 | 3 | 4 | 5 |
| RC_18 Sometimes I find I am still struggling to deal with my problem(s) or to change my situation. | 1 | 2 | 3 | 4 | 5 |
| RC_19 I wish I had more ideas on how to solve my problems or change my situation. | 1 | 2 | 3 | 4 | 5 |
| RC_20 I have started working on my problem or changing my situation, but I would like help. | 1 | 2 | 3 | 4 | 5 |
| RC_21 Maybe someone or something will be able to help me. | 1 | 2 | 3 | 4 | 5 |
| RC_22 I may need a boost right now to help me maintain the changes I have already made. | 1 | 2 | 3 | 4 | 5 |
| RC_23 I may be part of the problem, but I don’t really think I am. | 1 | 2 | 3 | 4 | 5 |
| RC_24 I hope that someone will have some good advice for me. | 1 | 2 | 3 | 4 | 5 |
| RC_25 Anyone can talk about changing; I’m actually doing something about it. | 1 | 2 | 3 | 4 | 5 |
| RC_26 Why can’t people just forget their problems? | 1 | 2 | 3 | 4 | 5 |
| RC_27 I’m struggling to improve myself after having a relapse of my problems or returning to a situation I know was bad for me. | 1 | 2 | 3 | 4 | 5 |
| RC_28 It is frustrating, but I feel I might be having a reoccurrence of a problem I thought I resolved. | 1 | 2 | 3 | 4 | 5 |
| RC_29 I have worries, but so does everyone. | 1 | 2 | 3 | 4 | 5 |
| RC_30 I am actively working on my problem or trying to change a negative situation. | 1 | 2 | 3 | 4 | 5 |
| RC_31 I would rather cope with my problems or situation than try to change them. | 1 | 2 | 3 | 4 | 5 |
| RC_32 After all I have done to try and change my problem or situation, comes back to haunt me every now and again. | 1 | 2 | 3 | 4 | 5 |

***Qualitative questions***

“I am now going to ask you some questions and there are no choices like in the previous questions. These questions are for us to learn about how you heard about the Exit Doors Here program and came to be enrolled in it, what your experience is so far, and what you hope to gain from the program. There are no right or wrong answers. We are most interested in hearing about your experience in your own words”.

**I_Q01 How did you first hear about the Elizabeth Fry Exit Doors Here program?**

[*prompt:* Did someone tell you about the program? Did you see a poster advertising the program?]

**I_Q02 What would you say are the main reasons for enrolling in the Exit Doors Here**

**program?**

**I_Q03 Was there anything that made you hesitate about joining the Exit Doors Here**

**program when you were first told about it?**

**I_Q04 Can you please describe what your experience has been like while enrolled in the**

***Exit Doors Here* program?**

**I_Q05 What knowledge and/or supports do you currently have in place to help**

**you make changes in your life or in your focus areas for the program?**

**I_Q06 What knowledge and/or supports are you hoping to gain from the *Exit Doors***

***Here* program which will help you make changes in your life or your focus areas**

**for the program?**

***Socio-Demographic Questions***

“We are almost done, thank you for your patience! I would now like to ask you some general socio-demographic questions. We are asking these questions so we can have an overall picture of the program’s participants. You do not have to answer any question that makes you feel uncomfortable”.

**SD_Q01 How old are you? Are you...**

☐18-24

☐25-34

☐35-44

☐45-54

☐55-64

☐65 and over

☐Decline to answer

**SD_Q02 Were you born in Canada?**

☐Yes

☐No

☐Decline to answer

**SD_Q03 If no, where were you born?**

**SD_Q04 People in Canada come from many racial and cultural groups. You may belong**

**to more than one group on the following list. Are you:**

☐White

☐Chinese

☐South Asian (e.g. Sri Lankan, Pakistani, East Indian, etc.)

☐Black

☐Filipino

☐Latin American

☐South East Asian (e.g. Vietnamese, Cambodian, Laotian, Thai, etc.)

☐Arab

☐West Asian (e.g. Iranian, Afghan, etc.)

☐Japanese

☐Korean

☐Aboriginal, that is, First Nations (North American Indian), Metis or Inuk (Inuit)

☐Don’t know

☐Other (please specify)

☐Decline to answer

**SD_Q05 What is your current relationship status?**

☐Single

☐Dating

☐Long-term relationship

☐Married/common-law partner

☐Separated/divorced

☐Never married

☐Widowed

☐Decline to answer

**SD_Q06 Do you have any children or dependents?**

☐Yes **(If yes, how many?** _______ )

☐No

☐Decline to answer

**SD_Q07 What is the highest level of education you have completed?**

☐Grade 8 or below

☐Grade 9-10

☐Grade 11-13

☐Completed High School

☐Trades certificate, diploma of vocational school or apprenticeship training (for example, hairstyling, carpeting, electrician)

☐College certificate or diploma

☐University certificate or diploma below Bachelor level

☐University degree at Bachelor level

☐University degree above Bachelor level

☐Decline to answer

“Thank you for taking part in our study and for answering our questions! We will contact you about one month after you finish the *Exit Doors Here* program to hear about your experience with the program. We would like to offer you a $50 gift card as thanks for talking with us today.”

**Additional Notes**
