# Supplementary material for: Helping women transition out of sex work: study protocol of a mixed-methods process and outcome evaluation of a sex work exiting program
Source: BMC Womens Health. 2020 Oct 9;20:227. doi: 10.1186/s12905-020-01086-3 (PMC7545381; doi:10.1186/s12905-020-01086-3)
Supplement: Supplementary file 2 — Additional file 2. [file 12905_2020_1086_MOESM2_ESM.docx]

**CONFIDENTIAL**

**EXIT DOORS HERE PROGRAM EVALUATION**

**POST- NTERVENTION QUESTIONNAIRE**

**Date:**

**Participant ID:**

**CONFIDENTIAL**

**“**Thank you again for agreeing to take part in this survey. We will be asking some questions, as we did at the beginning of this program, to learn about your experience with the *Exit Doors Here* program and the progress you made while you were enrolled in the program. We will also ask a few questions so we can have an overall picture of the program’s participants. Remember that there is no right or wrong answer, and you can skip any question you do not feel comfortable answering. This survey should take about one hour to complete. Please be assured that your name will not be linked to any information you provide.”

*Housing Situation & Goals*

“I am first going to ask you some questions about your housing situation and any plans you might have about your housing situation in the future”.

**H_ Q01 What is your current living arrangement?**

☐Renting a house/apartment/condominium

☐Own a house/condominium

☐Renting a room in a house/apartment with roommates

☐Don’t have a place to live.

☐Staying temporarily with family members or friends

☐Staying at an emergency shelter

☐Staying at a women’s hostel

☐Decline to answer

☐Other ____________________________

**H_Q02 How satisfied are you in your current living situation?**

☐Very satisfied

☐Satisfied

☐Somewhat satisfied

☐Neither satisfied nor dissatisfied (neutral)

☐Somewhat dissatisfied

☐Dissatisfied

☐Very dissatisfied

☐Decline to answer

**H_Q03 Since starting the Exit Doors Here program, what changes have you been able to**

**make with regards to your housing situation? (probe: when you started this program, did you have any plans related to your housing situation?).**

**H_Q04 Going forward, do you have any plans related to your housing situation? (Check**

**all that apply).**

☐I would like to move into a new apartment/home

☐I want my own place to live

☐I want to live in housing that I can afford

☐I do not want to live with my family members/roommates anymore

☐I want to feel safer in my home

☐I want to live in a safer neighbourhood

☐I want to move into an apartment/home that is more spacious

☐I need housing that is accessible for a physical disability

☐I want a place to live that I feel proud of

☐I want my landlord to fix the repairs needed in my apartment/home

☐I would like to live closer to my friends/family

☐I am satisfied with my current housing situation, I do not plan to change it

☐Other (please specify)

☐Decline to answer

**H_Q05 If you want to change your housing situation but can’t, what are the reasons?**

**(Check all that apply)**

☐I can’t afford to move/can’t find an affordable place to live

☐I am scared to leave my current situation

☐I need to keep living near a family member (e.g., a parent in a nursing home)

☐I need to live near my children’s school/daycare

☐I need to find a new job, so I can afford to move

☐I am awaiting subsidized housing/ benefits

☐I have experienced discrimination from potential landlords when trying to look for a new place to live

☐Other (please specify)

☐Decline to answer

***Employment, Educational & Financial Situation & Goals***

“I am now going to ask you questions about your employment and financial situation, and any plans you might have for them. You can skip any question you do not want to answer”.

**E_Q01 Are you employed?**

☐Yes

☐No **🡪 Skip to E_Q03**

☐Decline to answer

**E_Q02** [**If employed**], how satisfied are you in your current employment position?

☐Very satisfied

☐Satisfied

☐Somewhat satisfied

☐Neither satisfied nor dissatisfied (neutral)

☐Somewhat dissatisfied

☐Dissatisfied

☐Very dissatisfied

☐Decline to answer

**E_Q03 What was your individual income last year, before tax?**

☐$15,000 or under

☐$15,001-$25,000

☐$25,001-$35,000

☐$35,001-$50,000

☐$50,001-$75,000

☐More than $75,000

☐Decline to answer

**E_Q04 Since starting the Exit Doors Here program, what changes have you been able to**

**make with regards to your employment? (probe: when you started this program,**

**did you have any plans related to your employment?).**

**E_Q05 Do you have any plans to change jobs or receive further**

**education in the future? If so, what are they? (Check all that apply)**

☐I want to find a job

☐I want to find a different job

☐I want to train or become certified in a different field of employment

☐I want to enroll in college/university

☐I am satisfied with my current arrangement, I do not plan to change it

☐Other (please specify)

☐Decline to answer

**E_Q06 If you want to change jobs or receive further education but can’t, what are the**

**reasons? (Check all that apply)**

☐I don’t know where to start looking for information

☐I don’t have proper means of transportation

☐I don’t have proper childcare

☐I don’t have the skills or education to make the change

☐I have health issues that keep me from it

☐I have a learning disability

☐I have a permanent physical disability

☐Other (please specify)

☐Decline to answer

**E_Q07 Since starting the Exit Doors Here program, what changes have you been able to**

**make with regards to your finances? (probe: when you started this program,**

**did you have any plans related to your finances?).**

**E_Q08 Do you have any financial plans for the future? (Check all that apply)**

☐I want to earn more money

☐I want to become financially independent

☐I want to apply for social assistance and/or disability support

☐I want to open a bank account

☐I want to pay off my debt

☐I am satisfied with my current financial arrangement, I do not plan to change it

☐Other (please specify)

☐Decline to answer

**E_Q09 If you want to change your financial situation but can’t, what are the reasons?**

**(Check all that apply)**

☐I’m having trouble finding a job

☐I don’t know where to start looking for information

☐I don’t have the required skills/ education

☐I have health issues

☐Other (please specify)

☐Decline to answer

***Legal Issues***

“I am now going to ask you some questions and there are no choices like in the previous questions. These questions are for us to learn about any legal issue(s) you might be having and how it impacts your life.”

**L_Q01 Did you have any legal issue(s) when you started the Exit Doors Here program?**

(Prompt: Are you involved in any legal cases due to involvement of close family members? Examples of legal issues could be family court for child custody, or housing and tribunal, or working towards pardon, or diversion program, e.g. probation, volunteering, community health service, or witness to cases i.e. witness services)

**L_Q02 If so, what kind(s) of legal issue(s) do you have?**

**L_Q03 Have these legal issues changed in any way while you have been enrolled in the Exit Doors Here program?**

***Social Support Networks (Multidimensional Scale of Perceived Social Support (MSPSS))***

“When we first met when you started the *Exit Doors Here* program, we asked you to complete a questionnaire about your readiness to make changes in your life. I am now going to ask you the same questions again. I am going to read 11 statements to you. Some of the statements you might agree with, and others you might not. I would like you to tell me on a scale of 1-7 if you: 1=very strongly disagree; 2=strongly disagree; 3=mildly disagree; 4=neutral; 5=mildly agree; 6=strongly agree; 7=very strongly agree.” *(Interviewer hands scale to participant)*

| **Statement (indicator)** | **Very Strongly Disagree** | **Strongly Disagree** | **Mildly Disagree** | **Neutral** | **Mildly Agree** | **Strongly Agree** | **Very Strongly Agree** |
| --- | --- | --- | --- | --- | --- | --- | --- |
| SS_01  There is a special person who is around when I am in need. | 1 | 2 | 3 | 4 | 5 | 6 | 7 |
| SS_02  There is a special person with whom I can share my joys and sorrows. | 1 | 2 | 3 | 4 | 5 | 6 | 7 |
| SS_03  My family really tries to help me. | 1 | 2 | 3 | 4 | 5 | 6 | 7 |
| SS_04  I have a special person who is a real source of comfort to me. | 1 | 2 | 3 | 4 | 5 | 6 | 7 |
| SS_05  My friends really try to help me. | 1 | 2 | 3 | 4 | 5 | 6 | 7 |
| SS_06  I can count on my friends when things go wrong. | 1 | 2 | 3 | 4 | 5 | 6 | 7 |
| SS_07  I can talk about my problems with my family. | 1 | 2 | 3 | 4 | 5 | 6 | 7 |
| SS_08  I have friends whom I can share my joys and sorrows. | 1 | 2 | 3 | 4 | 5 | 6 | 7 |
| SS_09  There is a special person in my life that cares about my feelings. | 1 | 2 | 3 | 4 | 5 | 6 | 7 |
| SS_10  My family is willing to help me make decisions. | 1 | 2 | 3 | 4 | 5 | 6 | 7 |
| SS_11  I can talk about my problems with my friends. | 1 | 2 | 3 | 4 | 5 | 6 | 7 |

***Readiness for Self-Change (University Rhode Island Change Assessment Scale)***

“When we met a few months back when you had just started the *Exit Doors Here* program, we asked questions about your readiness to make changes in your life. I am now going to ask you those questions again. I am going to read you short statements. I would like you to tell me if you 1=Strongly Disagree; 2=Disagree; 3=Undecided; 4=Agree; 5=Strongly Agree” with each statement. *(Interviewer hands scale to participant)*

| **Statement** | **Strongly**  **Disagree** | **Disagree** | **Undecided** | **Agree** | **Strongly**  **Agree** |
| --- | --- | --- | --- | --- | --- |
| RC_01 As far as I am concerned, I don’t have any problems or situations in my life that I want to change. | 1 | 2 | 3 | 4 | 5 |
| RC_02 I think I might be ready for some self-change. | 1 | 2 | 3 | 4 | 5 |
| RC_03 I am doing something about the problem or situation that has been bothering me. | 1 | 2 | 3 | 4 | 5 |
| RC_04 It might be worthwhile to work on my problems or to change a situation in my life. | 1 | 2 | 3 | 4 | 5 |
| RC_05 As far as I am concerned, I don’t have any problems or negative situations that need changing. | 1 | 2 | 3 | 4 | 5 |
| RC_06 I am not the one with a problem, so it doesn’t make sense for me to consider changing. | 1 | 2 | 3 | 4 | 5 |
| RC_07 I am doing some work on my problems or taking steps to change a negative situation in my life. | 1 | 2 | 3 | 4 | 5 |
| RC_08 I have been thinking that I might want to change something about myself or a situation that I am in. | 1 | 2 | 3 | 4 | 5 |
| RC_09 I have been successful in working on my problem or changing a negative situation, but I’m not sure I can keep up the effort on my own. | 1 | 2 | 3 | 4 | 5 |
| RC_10 At times my situation or problem is difficult to deal with, but I am working on it. | 1 | 2 | 3 | 4 | 5 |
| RC_11 Trying to change is pretty much a waste of time for me. | 1 | 2 | 3 | 4 | 5 |
| RC_12 I’m hoping that I will be able to understand myself better. | 1 | 2 | 3 | 4 | 5 |
| RC_13 There is really nothing I need to change. | 1 | 2 | 3 | 4 | 5 |
| RC_14 I am really working hard to change. | 1 | 2 | 3 | 4 | 5 |
| RC_15 I have a problem, and I really think I should work on it. | 1 | 2 | 3 | 4 | 5 |
| RC_16 I’m not following through with what I have already changed as well as I had hoped, but I am not going to give up. | 1 | 2 | 3 | 4 | 5 |
| RC_17 Even though I’m not always successful in changing my situation or my problems, I am at least working on changing. | 1 | 2 | 3 | 4 | 5 |
| RC_18 Sometimes I find I am still struggling to deal with my problem(s) or to change my situation. | 1 | 2 | 3 | 4 | 5 |
| RC_19 I wish I had more ideas on how to solve my problems or change my situation. | 1 | 2 | 3 | 4 | 5 |
| RC_20 I have started working on my problem or changing my situation, but I would like help. | 1 | 2 | 3 | 4 | 5 |
| RC_21 Maybe someone or something will be able to help me. | 1 | 2 | 3 | 4 | 5 |
| RC_22 I may need a boost right now to help me maintain the changes I have already made. | 1 | 2 | 3 | 4 | 5 |
| RC_23 I may be part of the problem, but I don’t really think I am. | 1 | 2 | 3 | 4 | 5 |
| RC_24 I hope that someone will have some good advice for me. | 1 | 2 | 3 | 4 | 5 |
| RC_25 Anyone can talk about changing; I’m actually doing something about it. | 1 | 2 | 3 | 4 | 5 |
| RC_26 Why can’t people just forget their problems? | 1 | 2 | 3 | 4 | 5 |
| RC_27 I’m struggling to improve myself after having a relapse of my problems or returning to a situation I know was bad for me. | 1 | 2 | 3 | 4 | 5 |
| RC_28 It is frustrating, but I feel I might be having a reoccurrence of a problem I thought I resolved. | 1 | 2 | 3 | 4 | 5 |
| RC_29 I have worries, but so does everyone. | 1 | 2 | 3 | 4 | 5 |
| RC_30 I am actively working on my problem or trying to change a negative situation. | 1 | 2 | 3 | 4 | 5 |
| RC_31 I would rather cope with my problems or situation than try to change them. | 1 | 2 | 3 | 4 | 5 |
| RC_32 After all I have done to try and change my problem or situation, comes back to haunt me every now and again. | 1 | 2 | 3 | 4 | 5 |

***Awareness of Community Services & Supports***

“I am now going to ask you questions to learn about your awareness of community services and supports. I am going to read you 9 statements. I would like you to tell me if you 1=Strongly Disagree; 2=Disagree; 3=Undecided; 4=Agree; 5=Strongly Agree” with each statement. *(Interviewer hands scale to participant)*

| **Statement (indicator)** | **Strongly Disagree** | **Disagree** | **Undecided** | **Agree** | **Strongly Agree** |
| --- | --- | --- | --- | --- | --- |
| CSS_01  The support I received from the Exit Doors Here program helped me know what help is available in the community & how to access it. | 1 | 2 | 3 | 4 | 5 |
| CSS_02  The support I received from the Exit Doors Here Program helped me to use programs and services I hadn’t used before. | 1 | 2 | 3 | 4 | 5 |
| CSS_03  My life has improved as a result of knowing where to find community resources. | 1 | 2 | 3 | 4 | 5 |
| CSS_04  The supports I received from the Exit Doors Here program helped me to know what programs, services, and resources are available. | 1 | 2 | 3 | 4 | 5 |
| ***Since becoming involved with the program:*** | | | | | |
| CSS_05  I have met people I trust and whom I would turn to for advice if I were having problems. | 1 | 2 | 3 | 4 | 5 |
| CSS_06  I am more aware of resources. | 1 | 2 | 3 | 4 | 5 |
| CSS_07  I am more aware of services in my community. | 1 | 2 | 3 | 4 | 5 |
| CSS_08  I have used services that I had not previously used before the Exit Doors here program. | 1 | 2 | 3 | 4 | 5 |
| CSS_09  The support I have received from resources in the community helped me to meet my goals. | 1 | 2 | 3 | 4 | 5 |
| CSS_10 The support I’ve received from resources in the community will be maintained now that I’ve completed the Exit Doors Here program. | 1 | 2 | 3 | 4 | 5 |

***Qualitative questions***

“We are now on the last part of the interview. I am going to ask you questions and there are no choices like in the previous questions. These questions are for us to learn about your experiences in the Exit Doors Here program. There are no right or wrong answers.
We are most interested in hearing about the experience in your own words”.

**I_QO1** **You know the program is about exiting sex work, what parts of the program did you find most helpful around sex work?**

**I_QO2** **What parts of the program were not as helpful? (Probe: information sessions, 1 on 1, links with services)**

**I_Q03 What impact do you think the Exit Doors Here program has had on your**

**involvement in sex work?**

(Probe: another phrase for sex work: go out on dates, dance in clubs for money, exchange sexual acts for rent, food, clothing, shelter to sleep, working in a massage parlour, go out on dates at certain times e.g. end of the month).

**I_Q04 How would you describe your level of involvement in sex work this past month?** (Probe: another phrase for sex work: go out on dates, dance in clubs for money, exchange sexual acts for rent, food, clothing, shelter to sleep, working in a massage parlour, go out on dates at certain times e.g. end of the month). Circle response.

1. Not involved at all
2. Involved rarely
3. Involved frequently

**I_Q05 What do you think makes it hard for women to exit sex work? (probe: housing, income, access to services etc.)**

(Probe: another phrase for sex work: go out on dates, dance in clubs for money, exchange sexual acts for rent, food, clothing, shelter to sleep, working in a massage parlour, go out on dates at certain times e.g. end of the month).

**I_Q06 What knowledge and supports did you get over the course of this program? (e.g. knowledge and supports can include building a community, access to TTC tokens, clothing and food banks etc.)**

**I_Q07 Now that you have completed the program, do you feel more**

**confident to make changes in your life?**

**I_Q08 Is there anything that would have/ could help you exit sex work that you did not get from the program?**

**If yes, what?**

(Probe: another phrase for sex work: go out on dates, dance in clubs for money, exchange sexual acts for rent, food, clothing, shelter to sleep, working in a massage parlour, go out on dates at certain times e.g. end of the month).

**I_Q9 Do you feel that working with your Case Manager helped you overcome obstacles for exiting sex work? If yes, how**

(Probe: another phrase for sex work: go out on dates, dance in clubs for money, exchange sexual acts for rent, food, clothing, shelter to sleep, working in a massage parlour, go out on dates at certain times e.g. end of the month).

(Probe: how was your overall relationship with your case manager?)

**I_Q10 What, if anything, do you think needs to change about the Exit Doors Here**

**program?**

**Thank you for taking part in our study and for answering our questions. We would like to offer you a $50 gift card as thanks for talking with us today.** *[Proceed to give the participant their gift card.]*

**Additional notes:**
